# Testing process predictions of models of risky choice: a quantitative model comparison approach

**DOI:** 10.3389/fpsyg.2013.00646

**Published:** 2013-09-27

**Authors:** Thorsten Pachur, Ralph Hertwig, Gerd Gigerenzer, Eduard Brandstätter

**Affiliations:** ^1^Center for Adaptive Rationality, Max Planck Institute for Human DevelopmentBerlin, Germany; ^2^Center for Adaptive Behavior and Cognition, Max Planck Institute for Human DevelopmentBerlin, Germany; ^3^Department of Psychology, Johannes Kepler University of LinzLinz, Austria

**Keywords:** risky choice, heuristics, process tracing, similarity, strategy selection

## Abstract

This article presents a quantitative model comparison contrasting the process predictions of two prominent views on risky choice. One view assumes a trade-off between probabilities and outcomes (or non-linear functions thereof) and the separate evaluation of risky options (*expectation models*). Another view assumes that risky choice is based on comparative evaluation, limited search, aspiration levels, and the forgoing of trade-offs (*heuristic models*). We derived quantitative process predictions for a generic expectation model and for a specific heuristic model, namely the priority heuristic (Brandstätter et al., 2006), and tested them in two experiments. The focus was on two key features of the cognitive process: acquisition frequencies (i.e., how frequently individual reasons are looked up) and direction of search (i.e., gamble-wise vs. reason-wise). In Experiment 1, the priority heuristic predicted direction of search better than the expectation model (although neither model predicted the acquisition process perfectly); acquisition frequencies, however, were inconsistent with both models. Additional analyses revealed that these frequencies were primarily a function of what Rubinstein (1988) called “similarity.” In Experiment 2, the quantitative model comparison approach showed that people seemed to rely more on the priority heuristic in difficult problems, but to make more trade-offs in easy problems. This finding suggests that risky choice may be based on a mental toolbox of strategies.

In human decision making research, there are two major views on how people decide when faced with risky options (see Payne, 1973; Lopes, 1995). According to the first view, people evaluate risky options in terms of their expectation, that is, the weighted (by probability) average of the options' consequences. Prominent theories of risky choice (both past and present) such as expected value (EV) theory, expected utility (EU) theory, and prospect theory (Kahneman and Tversky, 1979) all belong to the family of expectation models. According to the second view, people choose between risky options using heuristics. A heuristic is a cognitive strategy that ignores part of the available information and limits computation. The heuristics view acknowledges that the decision maker is bounded by limits in his or her capacity to process information (Simon, 1955) and therefore often relies on simplifying principles to reduce computational demands (e.g., Tversky, 1969; Coombs et al., 1970; Payne et al., 1993). Following this view, Brandstätter et al. (2006) proposed the priority heuristic as an alternative account for several classic violations of EU theory (see below), which have usually been explained by modifying EV theory but retaining the expectation calculus. Whereas expectation models assume the weighting and summing of all information, the priority heuristic assumes step-wise comparison processes and limited search (for a discussion, see Vlaev et al., 2011)^1^.

How well do these two views—expectation models vs. models of heuristics—fare in capturing how people choose between risky options? Payne and Venkatraman (2011) have pointed out that the traditional focus in economics and in much psychological research has been on *what* decisions are made rather than *how* they are made. They listed several benefits of a better understanding of the processes involved (see also Svenson, 1979; Einhorn and Hogarth, 1981; Berg and Gigerenzer, 2010): for instance, one of the most important findings in behavioral decision research—the dependency of people's choices on task and context variations (e.g., Payne, 1976; Thaler and Sunstein, 2008)—will be better understood based on process models that predict how the order of reasons and other task features influence a choice (Payne et al., 1993; Todd et al., 2012). Relatedly, the prediction of individual differences in decision making will be enhanced if their modeling is not restricted to the behavioral level, but encompasses the process level as well. Finally, having accurate process models is crucial for improving decision making (cf. Schulte-Mecklenbeck et al., 2011).

In order to investigate the relative merits of the expectation and the heuristics views in describing the cognitive processes, we suggest two important methodological principles. First, because all models are idealizations and inevitably deviate from reality, model tests should be comparative (e.g., Lewandowsky and Farrell, 2010). Comparative tests enable researchers to evaluate which of several models fares better in accounting for the data (Neyman and Pearson, 1933; see also Gigerenzer et al., 1989; Pachur, 2011). Second, in addition to testing qualitative model predictions, it can also be informative to test quantitative predictions (Bjork, 1973), thus increasing the models' empirical content (Popper, 1934/1968). In this article, we provide an illustration of how quantitative process predictions of competing models of risky choice can be derived and pitted against each other.

In the following, we describe the expectation and heuristic approaches to modeling risky choice, summarize previous process investigations—including evidence for expectation models and heuristics—and finally derive quantitative process predictions for a generic expectation model and for a specific heuristic model, the priority heuristic. These predictions are then pitted against each other in two experiments. To preview one of our major findings: The results of the process tests indicate that one frequently used process measure, namely acquisition frequencies (defined as the frequency with which different reasons are inspected), appears to be only weakly (if at all) linked to how much weight people put on the reasons. In additional analyses, we found that acquisition frequencies are instead a function of the similarity of the options in a problem (cf. Rubinstein, 1988; Mellers and Biagini, 1994).

## Two views to risky choice: expectation models vs. heuristics

Since the Enlightenment, a key concept for understanding decision making under risk has been that of mathematical expectation, which at the time was believed to capture the nature of rational choice (Hacking, 1975). Calculating the expectation of a risky option involves examining the options' consequences and their probabilities, as well as weighting (multiplying) each consequence with its probability. This view is implemented in EV theory as well as in EU theory (which assumes the same process as EV theory, but replaces objective monetary amounts with subjective values). The view that people make risky choices based on expectation has been embraced by both normative and descriptive theories of risky choice (e.g., Kahneman and Tversky, 1979; Tversky and Kahneman, 1992; Birnbaum and Chavez, 1997; Mellers, 2000). Henceforth, we refer to models in this tradition as *expectation models* (see Payne, 1973).

Although the most time-honored expectation models—EV theory and EU theory—were soon found to be descriptively wanting, model modifications were proposed that are able to accommodate people's behavior while maintaining the core of expectation models (for an overview, see Wu et al., 2004)—for instance, (cumulative) prospect theory (Kahneman and Tversky, 1979; Tversky and Kahneman, 1992), the transfer-of-attention-exchange model (Birnbaum and Chavez, 1997), and decision-affect theory (Mellers, 2000). Expectation models have sometimes been interpreted as being mute as regards the processes underlying choice (e.g., Edwards, 1955; Gul and Pesendorfer, 2005). When taken at face value, however, they do have process implications that can be and have been spelled out (e.g., Russo and Dosher, 1983; Brandstätter et al., 2008; Cokely and Kelley, 2009; Glöckner and Herbold, 2011; Su et al., 2013). At the very least, expectation models imply two key processes: weighting and summing. Payne and Braunstein (1978) described the weighting (multiplication) and summing (adding) core of EV as follows:

Each gamble in a choice set is evaluated separately. For each gamble, the probability of winning and the amount to win are evaluated (multiplicatively) and then the probability of losing and the amount to lose are evaluated (multiplicatively), or vice versa. The evaluations of the win and lose components of the gamble are then combined into an overall value using an additive rule, or some simple variant. (p. 554).

Although modifications of EV theory such as prospect theory have introduced psychological variables such as reference points and subjective probability weighting, all of these modifications retain EV theory's assumption that human choice can or should be modeled based on the exhaustive weighting and summing processes that give rise to a compensatory decision process (e.g., in which a low probability of winning can be compensated by a high possible gain).

An alternative view of risky choice starts with the premise that people often do not process the given information exhaustively, but rely on simplifying heuristics (Savage, 1951; Tversky, 1969; Payne et al., 1993). Indeed, there is considerable evidence for people's use of heuristics in inferences under uncertainty (e.g., Pachur et al., 2008; García-Retamero and Dhami, 2009; Bröder, 2011; Gigerenzer et al., 2011; Pachur and Marinello, 2013), in decisions under certainty (e.g., Ford et al., 1989; Schulte-Mecklenbeck et al., in press), as well as in decisions under risk (e.g., Slovic and Lichtenstein, 1968; Payne et al., 1988; Cokely and Kelley, 2009; Venkatraman et al., 2009; Brandstätter and Gussmack, 2013; Pachur and Galesic, 2013; Su et al., 2013). This evidence is consistent with the argument that people find trade-offs—the very core of expectation models—difficult to execute, both cognitively and emotionally (Hogarth, 1987; Luce et al., 1999).

Many (but not all) heuristics forego trade-offs. One class of heuristics escapes trade-offs by statically relying on just one reason (attribute, cue). The minimax heuristic is an example: it chooses the option with the better of the two worst outcomes, ignoring its probabilities as well as the best outcomes (Savage, 1951). A second class of heuristics processes several reasons in a lexicographic order (Menger, 1871/1990). Unlike minimax, these heuristics search through several reasons, stopping at the first reason that enables a decision (Fishburn, 1974; Thorngate, 1980; Gigerenzer et al., 1999). The *priority heuristic* (Brandstätter et al., 2006), which is related to lexicographic semi-orders (Luce, 1956; Tversky, 1969), belongs to this class. Its processes include established psychological principles of bounded rationality (see Gigerenzer et al., 1999), such as sequential search, stopping rules, and aspiration levels. The priority heuristic assumes that probabilities and outcomes are compared between options, rather than integrated within options (as the weighting and summing operations suggest). For choices between two-outcome options (involving only gains), the priority heuristic proceeds through the following steps:

### Priority rule

Go through reasons in the order of: minimum gain, probability of minimum gain, and maximum gain.

### Stopping rule

Stop examination if the minimum gains differs by 1/10 (or more) of the maximum gain; otherwise, stop examination if probabilities differ by 1/10 (or more) of the probability scale. (To estimate the aspiration level, numbers are rounded up or down toward the nearest prominent number; see Brandstätter et al., 2006).

### Decision rule

Choose the option with the more attractive gain (probability).

For losses, the heuristic remains the same, except that “gains” are replaced by “losses.” The heuristic has also been generalized to choice problems with more than two outcomes (with the probability of the maximum outcome being included as the fourth and final reason) and to mixed gambles (see Brandstätter et al., 2006).

Due to its stopping rule, the priority heuristic terminates search after one, two, or three reasons (see the priority rule), depending on the choice problem. Henceforth, we will refer to choice problems where the heuristic stops after one, two, or three reasons as *one-reason choices, two-reason choices*, and *three-reason choices*, respectively (see Johnson et al., 2008).

## Empirical evidence for expectation models and heuristics in risky choice

How successful are the two views—models in the expectation tradition and heuristics—in capturing how people make risky choices? Expectation models have been successful in accounting for several established phenomena in people's overt choices (e.g., Kahneman and Tversky, 1979; but see Birnbaum, 2008b). For instance, they can account for classic violations of EV theory and EU theory, such as the certainty effect, the reflection effect, the fourfold pattern, the common consequence effect, and the common ratio effect. Moreover, they have proved useful in mapping individual differences (Pachur et al., 2010; Glöckner and Pachur, 2012).

Nevertheless, when researchers turned to examining the processes underlying risky choice, the common conclusion was that people do not comply with the process predictions of expectation models. For instance, “search traces in general were far less complex than would be expected by normative models of decision making. Instead, we found many brief search sequences” (Mann and Ball, 1994, p. 135; for similar conclusions, see Payne and Braunstein, 1978; Russo and Dosher, 1983; Arieli et al., 2011; Su et al., 2013). Moreover, whereas expectation models predict that transitions should occur mainly between reasons within an option (to compute its expectation), empirical findings have shown that transitions between options and across reasons are rather balanced and that the latter are sometimes even more prevalent—indicative of heuristic processes (Rosen and Rosenkoetter, 1976; Payne and Braunstein, 1978; Russo and Dosher, 1983; Mann and Ball, 1994; Lohse and Johnson, 1996). In addition, past research often observed variability across gamble problems in the amount of information examined, which has also been interpreted as hints at people's use of non-compensatory heuristics (e.g., Payne and Braunstein, 1978; Russo and Dosher, 1983; Mann and Ball, 1994; cf. Slovic and Lichtenstein, 1968). In a recent eye-tracking investigation of risky choice, Su et al. (2013) observed that people's information acquisition patterns deviated strongly from those found when they followed a weighting-and-adding process and were instead more in line with a heuristic process.

Consistent with these findings, several analyses have provided support for the priority heuristic as a viable alternative to expectation models. First, it has been shown that the priority heuristic logically implies several classic violations of EU theory—including the common consequence effect, common ratio effects, the reflection effect, and the fourfold pattern of risk attitude (see Katsikopoulos and Gigerenzer, 2008, for proofs). In addition, Brandstätter et al. (2006) showed that the priority heuristic can account for the certainty effect (Kahneman and Tversky, 1979) and intransitivities (Tversky, 1969). Second, across four different sets with a total of 260 problems, the priority heuristic predicted the majority choice better than each of three expectation models (including cumulative prospect theory) and ten other heuristics (Brandstätter et al., 2006). Further, in verbal protocol analyses Brandstätter and Gussmack (2013) found that people most frequently mentioned the reason that determines the choice according to the priority heuristic.

Nevertheless, several studies have also found clear evidence conflicting with the predictions of the priority heuristic (e.g., Birnbaum and Gutierrez, 2007; Birnbaum, 2008a; Birnbaum and LaCroix, 2008; Rieger and Wang, 2008; Rieskamp, 2008; Ayal and Hochman, 2009; Glöckner and Herbold, 2011). Fiedler (2010), for instance, observed that people's preferences between options were sensitive to information that according to the priority heuristic should be ignored. Moreover, Glöckner and Pachur (2012) reported that the priority heuristic was outperformed by cumulative prospect theory in predicting individual choice (rather than majority choice, as in Brandstätter et al., 2006). Furthermore, it has been argued that people do not prioritize their attention in the way predicted by the priority heuristic (Glöckner and Betsch, 2008a; Hilbig, 2008). Based on a fine-grained process analysis, Johnson et al. (2008) reported 28 tests of the priority heuristic; 11 were in the direction predicted by the heuristic, whereas 3 were in the opposite direction and 14 were not significant (see their Tables 1 and 2 on p. 268 and p. 269, respectively). From this result, Johnson et al. concluded that the priority heuristic fails to predict major characteristics of people's acquisition behavior.

What do these findings mean for the heuristics view of risky choice? Many authors reporting findings inconsistent with the predictions of the priority heuristic have concluded that people follow a compensatory mechanism (e.g., Johnson et al., 2008; Rieskamp, 2008; Ayal and Hochman, 2009; Glöckner and Herbold, 2011; but see Fiedler, 2010)—even though authors such as Slovic and Lichtenstein (1971) long ago concluded that people “have a very difficult time weighting and combining information” (p. 724). Importantly, however, only few previous process tests of the priority heuristic have directly compared the priority heuristic with the predictions of a compensatory mechanism (Brandstätter et al., 2008; Glöckner and Herbold, 2011). Moreover, as no model can capture psychological processes perfectly, the question is not so much whether a precise process model deviates from the observed data—it always will—but how large the deviation is relative to an alternative model. Therefore, the priority heuristic and expectation models should also be tested in a quantitative model comparison (Lewandowsky and Farrell, 2010). To make progress toward this goal, we next demonstrate how quantitative process predictions can be derived from the priority heuristic and expectation models and then test them against each other^2^.

## Modeling risky choice: quantitative process predictions

Previous investigations of the cognitive processes underlying risky choice have rarely derived quantitative predictions for different models and tested them comparatively (for an exception, see Payne et al., 1988). Instead, process data have been related to existing models in a qualitative rather than quantitative fashion, focusing on relatively coarse differences (such as reason-wise or gamble-wise information search, and compensatory or non-compensatory information processing; e.g., Rosen and Rosenkoetter, 1976; Ford et al., 1989; Mann and Ball, 1994; Su et al., 2013). It is only recently that process data have been directly used to test specific models of risky choice (Johnson et al., 2008), and few investigations have pitted the predictions of several models against one another (Brandstätter et al., 2008; Glöckner and Herbold, 2011).

What are the process implications of expectation models and the priority heuristic? The deliberate determination of an expectation requires weighting and summing processes of all information, as described by Payne and Braunstein (1978; see above). This holds across all models that have an expectation core; in this article, we therefore compare the process predictions of a generic expectation model against those of the priority heuristic. The priority heuristic does not weigh and sum, but assumes a sequential search process that is stopped once an aspiration level is met. The key differences between the priority heuristic and the expectation model can be operationalized in terms of two commonly examined features of cognitive search: frequency of acquisition and direction of search. [In the following analysis, we consider the priority heuristic without the preceding step of trying to find a no-conflict solution (Brandstätter et al., 2008). Including that step would require auxiliary assumptions about cognitive processes that need to be based on evidence. This evidence is currently not available]. We measure both features of cognitive search using the widely used process-tracing methodology Mouselab (Payne et al., 1993; Willemsen and Johnson, 2011). Information about the options (i.e., outcomes and probabilities) is concealed behind boxes on a computer screen, but can be rendered visible by clicking on those boxes. As a cautionary note, we should emphasize that current process models of risky choice are underspecified with regard to the memory, motor, and attention processes. Therefore, the predictions derived here are based on simplifications and should be regarded as a first step toward a complete account of the cognitive processes involved.

### Frequency of acquisition

The frequency of acquisition of a reason is measured as the number of times people inspect the information (e.g., by opening the respective box in Mouselab). To derive quantitative predictions, we assumed for all models an initial reading phase during which all boxes are examined once. Such an initial reading phase, in which the stimuli are encoded, is a common assumption in models of risky choice (e.g., Kahneman and Tversky, 1979; Goldstein and Einhorn, 1987). We calculated for each reason (e.g., minimum gains, probability of minimum gains) the relative frequency of acquisitions: the absolute number of acquisitions as predicted by the expectation model and the priority heuristic, respectively, divided by the total number of acquisitions, separately for one-, two-, and three-reason choices. As we collapsed across gain and loss problems, maximum gains and maximum losses will be referred to as “maximum outcomes,” and minimum gains and minimum losses as “minimum outcomes.” The predicted acquisition frequencies for each reason are shown in Appendix A. For instance, the priority heuristic predicts that 20% of all acquisitions in one-reason choices apply to the maximum outcomes (all of which are due to the reading phase), relative to 40% to the minimum outcomes. The expectation model, in contrast, predicts that the acquisition frequencies for the two reasons—or, more generally, for all reasons—are the same (25%; see Brandstätter et al., 2008; Glöckner and Herbold, 2011). As found by Su et al. (2013), people indeed inspect all information equally frequently when following an expectation-based strategy. The priority heuristic predicts five systematic deviations from this uniform distribution of acquisition frequencies (Table 1). Here, we focus on those following directly from the priority heuristic's stopping rule.

**Table 1:** **Tests of the relative acquisition frequencies predicted by the Priority Heuristic (PH) and modifications of expected utility theory (Expectation Model; EM) in Experiment 1**.

**Acquisition frequencies compared**	**Prediction**		**Data**		**Model supported**
	**PH**	**EM**	***M*s (%)**	**Test statistic**	
*O* vs. *P*	*O*_*r* = 1_ > *P*_*r* = 1_	*O*_*r* = 1_ = *P*_*r* = 1_	57.1 > 42.9	*t*_(479)_ = 12.7, *p* = 0.001	Priority heuristic
	*O*_*r* = 3_ > *P*_*r* = 3_	*O*_*r* = 3_ = *P*_*r* = 3_	55.5 > 44.5	*t*_(239)_ = 6.8, *p* = 0.001	Priority heuristic
*O*^max^ vs. *O*^min^	*O*^max^_*r* = 1_ < *O*^min^_*r* = 1_	*O*^max^_*r* = 1_ = *O*^min^_*r* = 1_	28.2 = 28.8	*t*_(479)_ = −0.81, *p* = 0.42	Expectation model
	*O*^max^_*r* = 2_ < *O*^min^_*r* = 2_	*O*^max^_*r* = 2_ = *O*^min^_*r* = 2_	30.3 > 25.2	*t*_(239)_ = 4.8, *p* = 0.001	Neither
*P*^max^ vs. *P*^min^	*P*^max^_*r* = 2_ < *P*^min^_*r* = 2_	*P*^min^_*r* = 2_ = *P*^max^_*r* = 2_	25.5 > 19.0	*t*_(239)_ = 4.7, *p* = 0.001	Neither
	*P*^max^_*r* = 3_ < *P*^min^_*r* = 3_	*P*^min^_*r* = 3_ = *P*^max^_*r* = 3_	24.2 > 19.6	*t*_(239)_ = 3.3, *p* = 0.001	Neither
*O*^max^_*r* = 1_, *O*^max^_*r* = 2_ vs. *O*^max^_*r* = 3_	*O*^max^_*r* = 1_ < *O*^max^_*r* = 3_	*O*^max^_*r* = 1_ = *O*^max^_*r* = 3_	28.2 < 31.2	*t*_(718)_ = −3.8, *p* = 0.001	Priority heuristic
	*O*^max^_*r* = 2_ < *O*^max^_*r* = 3_	*O*^max^_*r* = 2_ = *O*^max^_*r* = 3_	30.3 = 31.2	*t*_(478)_ = −1.0, *p* = 0.30	Expectation model
*P*^min^_*r* = 1_ vs. *P*^min^_*r* = 2_, *P*^min^_*r* = 3_	*P*^min^_*r* = 1_ < *P*^min^_*r* = 2_	*P*^min^_*r* = 1_ = *P*^min^_*r* = 2_	22.7 > 19.0	*t*_(718)_ = 3.5, *p* = 0.001	Neither
	*P*^min^_*r* = 1_ < *P*^min^_*r* = 3_	*P*^min^_*r* = 1_ = *P*^min^_*r* = 3_	22.7 > 19.6	*t*_(718)_ = 2.9, *p* = 0.004	Neither

O, outcomes; P, probabilities. Maximum and minimum outcomes and their probabilities are denoted as O^max^, O^min^, P^max^, and P^min^, respectively. The rs in the subscripts refer to one- (r = 1), two- (r = 2), and three-reason (r = 3) choices.

First, the heuristic predicts that, in one- and three-reason choices, outcomes are looked up more frequently than probabilities. More precisely, the relative acquisition frequencies for outcomes should be 60/40 = 1.5 times higher than for probabilities in one-reason choices, and 57.2/42.9 = 1.33 times higher in three-reason choices. Second, in one- and two-reason choices, the acquisition frequencies for the minimum outcomes are predicted to be higher (specifically, twice as high) than those for the maximum outcomes. The reason is that in one- and two-reason choices maximum outcomes are not examined after the reading phase. This also implies that, third, the relative acquisition frequencies for the maximum outcomes are predicted to be higher in three-reason than in one- and two-reason choices (1.4 and 1.7 times higher, respectively). Fourth, the acquisition frequencies for the probabilities of the minimum outcomes should be higher than those for the probabilities of the maximum outcomes in two- and three-reason choices (twice as high). This follows from the fact that whereas the probabilities of the minimum outcomes are looked up in two- and three-reason choices, the probabilities of the maximum outcomes are examined only in choice problems with more than two outcomes. Finally, the acquisition frequencies for the probabilities of the minimum outcomes are predicted to be higher in two- and three- than in one-reason choices (1.7 and 1.4 times higher, respectively).

Note that we did not consider the hypothesis *O*^min^_*r* = 1_ < *O*^min^_*r* = 3_ tested by Johnson et al. (2008; see their Table 2) because the priority heuristic in fact does not make that prediction. As one- and three-reason choices differ only in terms of the acquisitions (in the choice phase) of the maximum outcome and the probability of the minimum outcome, the priority heuristic predicts that the absolute acquisition frequencies for the minimum outcomes do not differ between one- and three-reason choices (as does the expectation model). For the relative number of acquisitions of the minimum outcome, the priority heuristic predicts a decrease across one-, two-, and three-reason choices, respectively (see Appendix A).

### Direction of search

Direction of search is defined by the sequence of transitions between subsequent acquisitions. The priority heuristic and the expectation model differ in their predictions of how search proceeds through the reasons. The priority heuristic searches sequentially in a particular order, compares the gambles on the respective reasons, and stops after one, two, or three reasons (depending on the structure of the choice problem). The expectation model, in contrast, looks up all information for each gamble and integrates them. Therefore, it predicts more transitions within each gamble than the priority heuristic. Table 2 lists both models' exact quantitative transition probabilities (separately for one-, two-, and three-reason choices), as derived by Brandstätter et al. (2008). As for the acquisition frequencies, an initial reading phase is assumed in which all boxes are examined once (the predictions in Table 2 are collapsed across the reading phase and the choice phase; see Appendix A for the derivation of the predictions in greater detail). The predictions are formulated in terms of the percentages of outcome-probability transitions (i.e., transitions from an outcome to its corresponding probability), other within-gamble transitions, and within-reason transitions (e.g., from the minimum outcome of Gamble A to the minimum outcome of Gamble B) that the priority heuristic and the expectation model, respectively, expect to occur.

**Table 2:** **Predicted and observed transition percentages for the reading and choice phases combined in Experiments 1 and 2 (for Experiment 2, percentages are given separately for easy/difficult problems)**.

	***r* = 1**	***r* = 2**	***r* = 3**
**OUTCOME-PROBABILITY TRANSITIONS**			
**Predictions**			
Priority heuristic	50	50	42
Expectation model	57	57	57
Random search	14.29	14.29	14.29
**Results**			
Experiment 1	36.2	37.5	35.4
Experiment 2	–	43.2/42.8	–
**OTHER WITHIN-GAMBLE TRANSITIONS**			
**Predictions**			
Priority heuristic	25	20	25
Expectation model	29	29	29
Random search	28.57	28.57	28.57
**Results**			
Experiment 1	19.0	19.4	17.2
Experiment 2	–	18.8/16.4	–
**WITHIN-REASON TRANSITIONS**			
**Predictions**			
Priority heuristic	25	30	33
Expectation model	14	14	14
Random search	14.29	14.29	14.29
**Results**			
Experiment 1	24.4	23.2	25.6
Experiment 2	–	18.9/21.7	–

See Appendix A for detailed description of the derivation. r = number of reasons inspected by the priority heuristic. Note that the observed transition percentages do not add up to 100 as participants also made transitions that were both between-reasons and between-gambles. Such transitions, which could, for instance, be due to noise, are not predicted by the models. For the derivations of the predictions under random search, it was assumed that transitions between all boxes were equally likely. This yielded 42.86% transitions that were both between-reasons and between-gambles.

As pointed out by Johnson et al. (2008), predictions about transition probabilities are sensitive to the assumptions made. Specifically, Brandstätter et al. (2008) made the simplifying assumption that people initially read each piece of information once, first for gamble A, then for gamble B. The alternative would be that information is always read from left to right, independently of how the gambles are presented. In additional analyses reported in Appendix B, we tested this alternative assumption and found that the performance of the expectation model and the priority heuristic decreased. Therefore, the original assumption is retained here.

**Figure 1:**
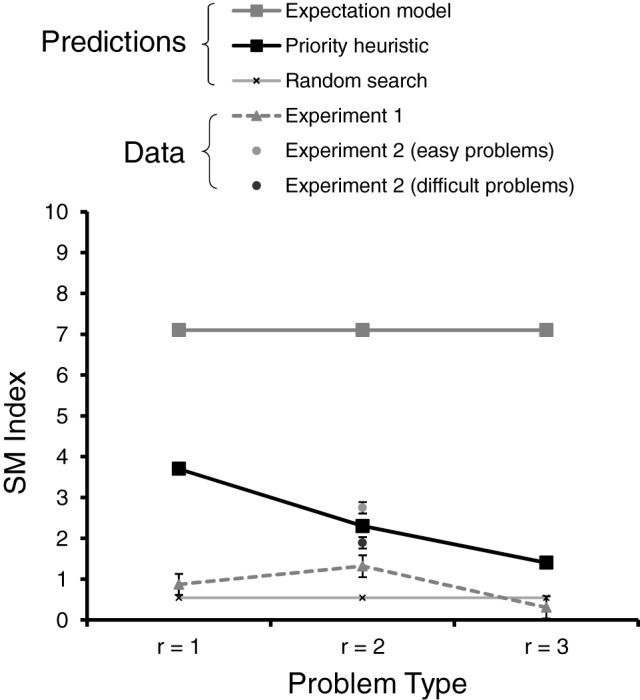
**Predicted and observed SM index (for reading and choice phases combined) in Experiments 1 and 2, separately for one-reason (*r* = 1), two-reason (*r* = 2), and three-reason (*r* = 3) choices**. The error bars represent standard errors of the mean.

We employed the search measure (SM) proposed by Böckenholt and Hynan (1994) to combine the transition percentages into an aggregate measure:

(1) $$SM=\frac{\sqrt{N}((GR/N)(n_{\text{gamble}}-n_{\text{reason}})-(R-G))}{\sqrt{G^{2}(R-1)+R^{2}(G-1)}},$$

where *G* is the number of gambles in a choice problem (two in our experiments), *R* is the number of reasons (four in our experiments), *N* is the total number of transitions, *n*_reason_ is the number of reason-wise transitions, and *n*_gamble_ is the number of gamble-wise transitions (see Appendix C for details). A negative value of SM indicates predominantly reason-wise search, and a positive value predominantly gamble-wise search. Figure 1 shows the predicted SM values for the expectation model (thick gray line) and the priority heuristic (thick black line), separately for one-reason, two-reason, and three-reason choices (also shown are the predictions under random search, to which we turn below). As can be seen, there are two SM predictions. First, the priority heuristic predicts systematically lower SM values (i.e., less gamble-wise processing) than does the expectation model. Second, the priority heuristic predicts SM values to decrease as more reasons are looked up: As more reasons are inspected, the contribution of the mainly gamble-wise reading phase to the overall direction of search decreases in relation to that of the mainly reason-wise choice phase.

In the following, we report two experiments that test these process predictions derived from the priority heuristic and the expectation model. In Experiment 1, participants were presented with “difficult” choice problems—that is, choice problems with options having similar expected values (we will define choice difficulty below). In Experiment 2, each participant was presented with both difficult and easy choice problems, allowing us to examine the hypothesis (Brandstätter et al., 2006; cf. Payne et al., 1993) that people use different strategies depending on characteristics of the environment.

## Experiment 1: how well do the priority heuristic and the expectation model predict process data?

### Methods

#### Participants

Forty students (24 female, mean age 27.4 years) from Berlin universities participated in the experiment, which was conducted at the Max Planck Institute for Human Development. Participants received a fixed hourly fee of €10. One of the gambles chosen by the participants was randomly selected, played out at the conclusion of the experiment and the average outcome was converted into a cash amount (with a factor of 10:1). On average, each participant received an additional amount of €4. Participants took around 55 min to complete the experiment.

#### Material

We used 24 binary choice problems consisting of two-outcome gambles (Appendix D). In each problem, the two gambles had similar expected values. Six of the 24 problems were taken from Kahneman and Tversky (1979), five from Brandstätter et al. (2006); the rest were constructed such that there were (i) the same number of gain and loss problems, and (ii) 12 of the 24 problems represented one-reason choices (i.e., problems for which the priority heuristic predicts that only the first reason will be looked up), 6 represented two-reason choices, and 6 represented three-reason choices.

#### Design and procedure

**Figure 2:**
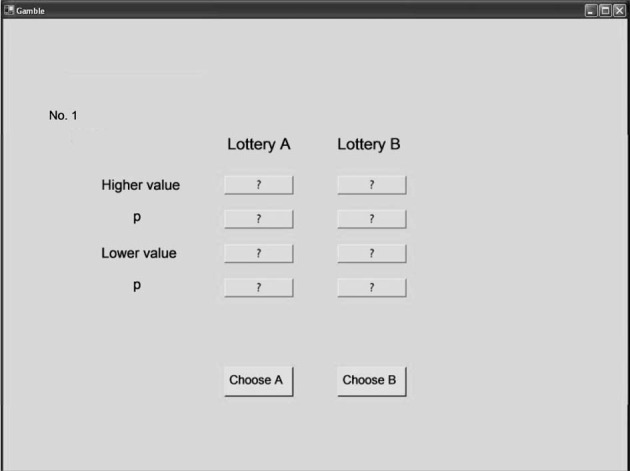
**Screenshot of the Mouselab program used in the experiments**.

In a programmed Mouselab task (Czienskowski, 2006), each participant was presented with the 24 choice problems one at a time and in randomized order. Information about the options (i.e., the four reasons) was concealed behind boxes (Figure 2). Labels placed next to the boxes indicated the type of information available, such as “higher value”, “lower value”, and “probability^3^.” For gain gambles, the higher and lower values were the maximum and minimum gains, respectively. For loss gambles, the higher and lower values were the minimum and maximum losses, respectively. Participants could open a box by clicking on it, and the information was visible for as long as the mouse was pressed. Participants were informed that they could acquire as much information as they needed to make a choice. The experimental protocol used can be found in Appendix E. We counterbalanced the different locations of the boxes on the screen across participants. Five participants were randomly assigned to each of eight presentation conditions (i.e., horizontal vs. vertical set-up × higher vs. lower value presented first × outcome information first vs. probability information first). There were no monetary search costs. Participants familiarized themselves with the Mouselab paradigm by performing nine practice trials. To examine the reliability of individual choice behavior, we presented participants with a subset of the gamble problems (see Appendix D) again, using a paper-and-pencil format. An interval of around 45 min separated the Mouselab and the paper-and-pencil tasks, during which participants performed an unrelated experiment.

### Results

In a first step, we examine the ability of the priority heuristic and the expectation model to predict people's choices. We then test the models' process predictions against the observed acquisition frequencies and direction of search.

#### Choices

Each individual's choices in those problems that were included in both the Mouselab and the paper-and-pencil tasks showed an average (Fisher transformed) correlation between the two measurements of *r* = 0.26, *t*_(39)_ = 4.61, *p* = 0.001 (one-sample *t*-test using the z-transformed individual *r*s). Note, however, that given that this analysis was based on only a subset of the problems used in the Mouselab task and different methods were used to collect people's preferences (computer vs. paper-and-pencil) this estimate of people's choice reliability might only be approximate. Next, we tested three expectation models [cumulative prospect theory (Tversky and Kahneman, 1992), security-potential/aspiration theory (Lopes and Oden, 1999), and the transfer-of-attention-exchange model (Birnbaum and Chavez, 1997)], and, in addition to the priority heuristic, 10 other heuristics (equiprobable, equal-weight, better-than-average, tallying, probable, minimax, maximax, lexicographic, least-likely, and least-likely; see Brandstätter et al., 2006, for a detailed description of each model). Following previous comparisons of expectation models and heuristics, we determined the proportion of choices correctly predicted by each model (e.g., Brandstätter et al., 2006; Glöckner and Pachur, 2012). As described in Appendix F, to derive the choice predictions of the expectation models we used parameter sets obtained in previous published studies, which is a common approach in the literature on risky choice (e.g., Brandstätter et al., 2006; Birnbaum and Bahra, 2007; Birnbaum, 2008b; Glöckner and Betsch, 2008a; Su et al., 2013)^4^. A more detailed description of the analysis and results can be found in Appendix F. The main result is that none of the three expectation models predicted individual choice better than the priority heuristic. Specifically, the priority heuristic achieved, on average (across participants), 62.6% correct predictions, somewhat better than the best expectation model, cumulative prospect theory (based on the parameter set by Tversky and Kahneman, 1992), at 58.9%, *t*_(39)_ = 2.34, *p* = 0.025 [both models' predictions were better than chance, *t*_(39)_ > 5.12, *p* < 0.001]^5^. The equiprobable and the equal-weight heuristics also achieved 58.9%; the transfer-of-attention-exchange model and security-potential/aspiration theory made 57.4% and 53.6% correct predictions, respectively.

#### Frequency of acquisition

On average, there were 12.7 (*SD* = 7.6) acquisitions per problem (or 1.6 per box)^6^. Inconsistent with the priority heuristic, the average (across gamble problems) number of acquisitions did not increase, but in fact decreased slightly across one-reason (*M* = 13.2), two-reason (*M* = 12.8), and three reason-choices (*M* = 11.9), *F*_(2, 959)_ = 2.35, *p* = 0.096. Note, however, that the effect was rather small, thus giving some support to the prediction of the expectation model (according to which the number of acquisitions should not be affected by problem type). Next, we determined for each reason its relative acquisition frequency (i.e., the percentage of acquisitions). To quantify the deviation of the models' predictions (Appendix A) from the observed acquisition percentages, we used the root mean squared deviation (RMSD), a simple and popular discrepancy measure (see Juslin et al., 2009; Lewandowsky and Farrell, 2010). Specifically, we calculated for each participant each model *k*'s RMSD between the observed relative frequency of acquisitions, *o*, and the prediction, *p*, of the model across all *N* (= 24) gamble problems and *J* (= 4) different reasons (note that, as in Johnson et al., 2008, we thus used the individual choice problems as the unit of analysis):

(2) $${\text{RMSD}}_{k}=\sqrt{\frac{\sum_{j\;=\;1}^{J}\sum_{n\;=\;1}^{N}{\left(o_{jn}-p_{jn,\;k}\right)}^{2}}{JN}}.$$

The average (across gamble problems) RMSD was lower for the expectation model than for the priority heuristic (indicating a lower discrepancy), *M*s = 9.8 vs. 12.5 (bootstrapped 95% confidence interval of the difference CI_diff_ = [−3.02, −2.48]), thus supporting the former^7^. Note that random search would make the same prediction as the expectation model, namely equal distribution across all reasons.

In addition, we tested the five directed predictions derived above concerning the relations of acquisition frequencies (see Table 1). Findings showed, for instance, that consistent with the priority heuristic's first prediction, outcomes were looked up more frequently than probabilities for one- and three-reason choices (ratio = 1.33 and 1.28, respectively). This focus on outcomes is inconsistent with the expectation model. Overall, participants more frequently acquired information about the maximum outcomes than about the minimum outcomes, *M*s = 29.5% vs. 27.0%, *t*_(959)_ = 4.77, *p* = 0.001. Consistent with the expectation model, but inconsistent with the priority heuristic's second prediction, the acquisition frequencies for the minimum outcomes in two- and three-reason choices were not higher than those for the maximum outcomes. Overall, few of either model's predictions were supported: in five out of ten cases, neither model was supported; in two cases, the expectation model was supported, and in three cases, the priority heuristic (see Table 1). Note again that random search would make the same predictions as the expectation model.

#### Direction of search

For each participant, we determined the percentage of transitions for the three predicted transition types—that is, how many transitions were an outcome-probability transition, a different type of within-gamble transition, or a within-reason transition. Predicted and mean actual percentages are shown in Table 2. The priority heuristic predicted the transition percentages consistently better than the expectation model. Each of the nine observed percentages (3 transition types × 3 problem types) was closer to the predictions of the priority heuristic than to those of the expectation model. To quantify the overall discrepancy between the observed, *o*, and the predicted transition percentages, *p*, for each model *k*, we calculated for each participant the RMSD across all *Q* (= 3) transition types and *M* (= 3) problem types:

(3) $${\text{RMSD}}_{k}=\sqrt{\frac{\sum_{q\;=\;1}^{Q}\sum_{m\;=\;1}^{M}{\left(o_{qm}-p_{qm,\;k}\right)}^{2}}{QM}}.$$

The priority heuristic showed a lower average (across participants) RMSD than did the expectation model, *M*s = 5.39 vs. 6.20, bootstrapped 95% CI_diff_ = [−1.48, −0.12], supporting the former. The priority heuristic showed a lower RMSD than a baseline model assuming random search, *M* = 6.84, bootstrapped 95% CI_diff_ = [−2.11, −0.82] (the predicted choice proportions under random search and details about their derivation can be found in Table 2). The expectation model's RMSD, by contrast, did not differ from the RMSD of the baseline model, bootstrapped 95% CI_diff_ = [−1.86, 0.56].

We next summarized the observed transition percentages using the SM index. There was no difference between the horizontal and the vertical set-ups of the boxes, *M* = 0.117, *SD* = 6.221 vs. *M* = 1.637, *SD* = 4.276, *t*_(33.68)_ = −0.901, *p* = 0.374. Figure 1 shows the average SM values separately for one-reason, two-reason, and three-reason choices (broken gray line), as well as the SM index assuming random search (thin gray line; based on the transition percentages under random search in Table 2). There are three key results. First, the observed values of the index were consistently lower than predicted by either the expectation model or the priority heuristic; in fact, they were relatively close to the prediction under random search, arguably resulting from noise in the acquisition process. Second, as can be seen from Figure 1, the values were clearly closer to the predictions of the priority heuristic than to those of the expectation model. Third, the direction of search differed between one-, two-, and three-reason choices, *F*_(2, 78)_ = 3.62, *p* = 0.031 (using a repeated-measures ANOVA with problem type as a within-subject factor), thus contradicting the expectation model and the pattern based on random search (which predicts an SM value of 0.54 irrespective of problem type). The priority heuristic, by contrast, predicts the direction of search to differ between one-, two-, and three-reason choices, though the linear trend predicted by the priority heuristic captured the pattern of SM values less accurately than did a quadratic trend, *F*_(1, 39)_ = 2.29, *p* = 0.139 vs. *F*_(1, 39)_ = 4.89, *p* = 0.033.

### Summary

We evaluated the expectation model and the priority heuristic in terms of their ability to predict two key features of the cognitive process. The picture provided by the tests of acquisition frequencies was inconclusive: Although the overall deviations between observed and predicted acquisition frequencies were smaller for the expectation model than for the priority heuristic, the tests of the ordinal predictions did not clearly favor one model over the other. We return to this issue shortly. The nine tests of direction of search (Table 2), by contrast, consistently supported the priority heuristic. Inconsistent with the expectation model, the direction of search as summarized in the SM index differed between one-, two-, and three-reason choices. Although the priority heuristic does predict the SM value to differ across problems types, it did not predict the observed pattern perfectly.

## Experiment 2: choices and processes in easy and difficult problems

We next apply the quantitative model comparison approach to investigate a central assumption of the adaptive toolbox view of risky choice (Payne et al., 1993; Brandstätter et al., 2008), namely, that strategy use is a function of the statistical characteristics of the environment (for support of this assumption in probabilistic inference, see, e.g., Rieskamp and Otto, 2006; Pachur et al., 2009; Pachur and Olsson, 2012). Specifically, we tested the hypothesis that different processes are triggered depending on the choice difficulty of a problem. Brandstätter et al. (2006, Figure 8; 2008, Figure 1) observed that how well various choice strategies can predict majority choice depends on the ratio of the expected values of the two options. This ratio can be understood as a proxy for the difficulty of the problem, with ratios between 1 and 2 representing “difficult problems” and ratios larger than 2 representing increasingly “easy problems.” As Brandstätter et al. (2008) pointed out, gaining a sense of how difficult a choice is does not require an explicit calculation of the expected values, but could be achieved, for instance, by a simple dominance check.

Brandstätter et al. (2006) found that several modifications of EU theory—security-potential/aspiration theory, cumulative prospect theory, the transfer-of-attention-exchange model, as well as the simplest expectation model, EV theory—predicted majority choice better for easy than for difficult problems. In contrast, the priority heuristic predicted majority choice better for difficult than for easy problems. This could mean that, as hypothesized by Brandstätter et al. (2006, 2008), easy problems elicit more trade-offs than difficult problems.

To test this hypothesis, we now compare the priority heuristic and cumulative prospect theory/EV theory (as explained below, the latter two always made the same prediction for the gamble problems used). Participants were presented with easy and difficult problems (using a within-subjects design); the problems were selected such that the priority heuristic and cumulative prospect theory predicted opposite choices. We therefore expected larger differences in the predictive abilities of the two models, relative to Experiment 1, in which the predictions of the two models often overlapped (in either 50% or 75% of the problems, depending on whether the parameter set of Erev et al. (2002), or Kahneman and Tversky (1979), is used for cumulative prospect theory). As in Experiment 1, we recorded participants' search behavior using the Mouselab methodology and compared the data to the process predictions of the priority heuristic and the expectation model (recall that on the process level, cumulative prospect theory and a generic expectation model imply the same weighting and summing processes).

### Methods

#### Participants

Forty students (28 female, mean age 24.4 years) participated in this experiment, which was conducted at the University of Basel. The payment schedule was very similar to Experiment 1 (i.e., participants received CHF 15 per hour, plus a bonus that was determined by their choices; one problem was randomly selected and the chosen gamble played out at the end of the session).

#### Material, design, and procedure

Each participant was presented with 48 choice problems, using a programmed Mouselab environment (the experimental protocol was very similar to that used in Experiment 1). Half of the problems represented difficult problems and the other half easy problems (based on the definition described above). Within easy and difficult problems, half were gain and half were loss problems. The problems were taken from Mellers et al. (1992). We sampled the problems as follows: First, we restricted the original set of 900 problems to those where the ratio of the gambles' expected values was between either 1 and 2 or 5 and 6. Next, we restricted the remaining problems to those in which the priority heuristic and cumulative prospect theory [irrespective of whether Tversky and Kahneman's (1992), or Lopes and Oden's (1999), parameter values were used] predicted opposite choices (for an example, see Appendix D). One hundred problems met these criteria, and for all problems cumulative prospect theory predicted the same choice as EV theory. We then randomly sampled from this set 24 gain problems—12 easy problems (with EV ratios between 5 and 6) and 12 difficult problems (with EV ratios around 1). Using the same constraints, we also sampled 24 loss problems (see Appendix D for a complete list of the problems). Note that in the Mellers et al. problem set, all minimum outcomes are zero, and the priority heuristic always based its choice on the second-ranked reason (i.e., the probability of the minimum outcomes). The forty-eight problems were presented in random order and participants were informed that they could acquire as much information as they needed to make a choice.

### Results

As in Experiment 1, we first examine participants' choices before analyzing the two process measures (i.e., acquisition frequency and direction of search).

#### Choices

**Figure 3:**
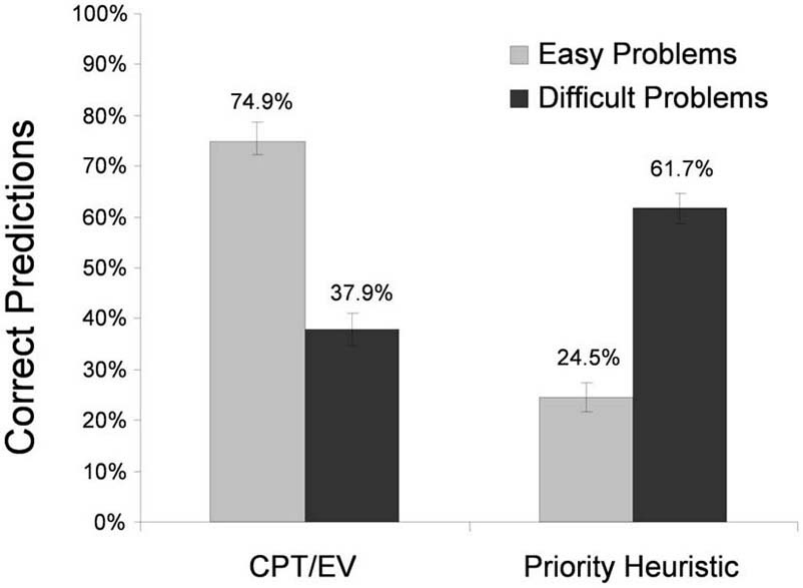
**Correct predictions of the individual choices in Experiment 2**. CPT, cumulative prospect theory, EV, expected value theory. The error bars represent standard errors of the mean.

Figure 3 shows the percentages of correctly predicted individual choices for the priority heuristic and cumulative prospect theory (and, as explained before, EV theory). Replicating Brandstätter et al.'s (2006, 2008) analyses, the expectation-based models—cumulative prospect theory and EV theory—predicted choices in easy problems much better than the priority heuristic did (*M* = 74.9%, *SE* = 2.7 vs. *M* = 24.5%, *SE* = 2.7 correct predictions). In contrast, the priority heuristic predicted choices in difficult problems markedly better than cumulative prospect theory and EV theory did (*M* = 61.7%, *SE* = 3.1 vs. *M* = 37.9%, *SE* = 3.1). In both easy problems and difficult problems, the predictions of the best-performing model were better than chance, *t*_(39)_ > 3.78, *p* < 0.001. The differential model performance between easy and difficult problems was corroborated statistically by a significant interaction (using a repeated-measures ANOVA) between choice difficulty (high vs. low) and model (priority heuristic vs. cumulative prospect theory/EV theory), *F*_(1, 39)_ = 112.63, *p* = 0.001^8^.

One interpretation of these results is that easy and difficult problems trigger different strategies. When problems are easy, participants tend to make choices consistent with expectation models, whereas when problems are difficult, they tend to make choices consistent with the priority heuristic. Our analysis of individual choices converges with Brandstätter et al.'s (2006, 2008) analyses of majority choices. Is there also process evidence for the use of different strategies in easy vs. difficult problems?

#### Frequency of acquisition

**Figure 4:**
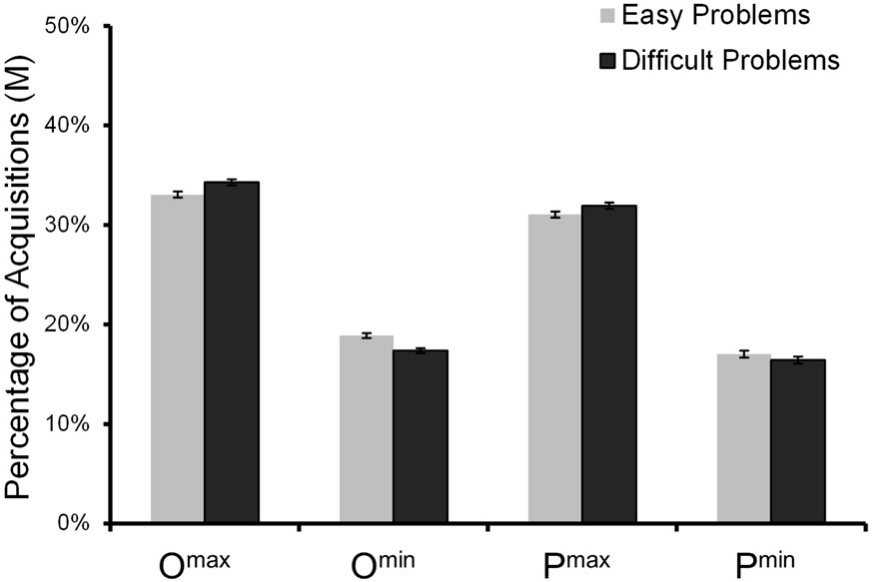
**Obtained relative acquisition frequencies for reading and choice phases combined in Experiment 2**. The error bars represent standard errors of the mean.

Across all eight boxes there were, on average, 14.3 (*SD* = 8.4) acquisitions (or 1.8 per box) before a choice was made (again, many fewer than in Johnson et al., 2008; see Footnote 6). Participants made fewer acquisitions in easy than in difficult problems, *M*s = 13.4 vs. 15.2, *F*_(1, 1916)_ = 26.4, *p* = 0.001. As in Experiment 1, we calculated for each reason its relative acquisition frequency (i.e., the percentage of acquisitions). Figure 4 shows the mean relative acquisition frequencies for each of the four reasons. Concerning the deviations of the predicted acquisition frequencies (see Appendix A) from the empirical ones, the expectation model showed, overall, a lower RMSD than did the priority heuristic, *M*s = 10.2 vs. 17.1, bootstrapped 95% CI_diff_ = [−6.99, −6.77]. This held for both easy problems (*M*s = 9.8 vs. 16.6, bootstrapped 95% CI_diff_ = [−7.00, −6.69]) and difficult problems (*M*s = 10.6 vs. 17.5, bootstrapped 95% CI_diff_ = [−7.07, −6.77]). Note that, like the expectation model, random search predicts an equal distribution of acquisitions across all reasons.

Experiment 2 included only choice problems for which the priority heuristic predicts that examination is stopped after the second reason (i.e., two-reason choices); therefore, only two of the five predictions in Table 1 can be tested. The results concerning the first prediction were inconsistent with both the priority heuristic and the expectation model: Maximum outcomes were looked up more frequently than minimum outcomes, *M*s = 33.6% vs. 18.1%, *F*_(1, 1916)_ = 2067.2, *p* = 0.001 (Figure 4). Likewise, the results concerning the third prediction were inconsistent with both the priority heuristic and the expectation model: the probabilities of the maximum outcomes were looked up more frequently than those of the minimum outcomes, *M*s = 31.5% vs. 16.7%, *F*_(1, 1916)_ = 1159.2, *p* = 0.001. Surprisingly, the qualitative pattern of the acquisition frequencies did not differ between easy and difficult problems, apparently at odds with the conclusion from the choices that people switch strategies between easy and difficult problems (Figure 4). We return to this issue shortly.

#### Direction of search

As in Experiment 1, we calculated for each participant and separately for difficult and easy problems the RMSD for each model. Consistent with the hypothesis that compensatory processes as represented by the expectation model are more likely to be triggered by easy than by difficult problems, the average RMSD for the expectation model was smaller for easy than for difficult problems, *M*s = 7.73 vs. 8.34, bootstrapped 95% CI_diff_ = [−0.99, −0.23]. Consistent with the hypothesis that a non-compensatory process is more likely to be triggered in difficult than in easy problems, the average RMSD for the priority heuristic was smaller for difficult than for easy problems, *M*s = 7.10 vs. 7.62, bootstrapped 95% CI_diff_ = [−0.96, −0.06]. Moreover, the priority heuristic had a smaller RMSD than the expectation model for the difficult problems (*M*s = 7.10 vs. 8.34, bootstrapped 95% CI_diff_ = [−2.44, −0.09]), but not for the easy ones (*M*s = 7.62 vs. 7.73, bootstrapped 95% CI_diff_ = [−1.29, 1.09]). For difficult problems, the mean RMSD expected under random search was 12.34, which was higher than the expectation model's (bootstrapped 95% CI_diff_ = [2.73, 6.13]) and the priority heuristic's RMSD (bootstrapped 95% CI_diff_ = [3.51, 5.69]). For easy problems, the mean RMSD expected under random search was 12.19, and also this was higher than the expectation model's (bootstrapped 95% CI_diff_ = [3.02, 6.25]) and the priority heuristic's RMSD (bootstrapped 95% CI_diff_ = [4.19, 6.30]).

There was no difference in direction of search, as indicated by the SM index, between the horizontal and vertical set-ups of the boxes, *M*s = 2.68 vs. 1.89, *t*_(39)_ = 0.56, *p* = 0.58. As Figure 1 shows, the SM index was smaller in difficult than in easy problems, *M*s = 1.89 vs. 2.75; *t*_(39)_ = −4.43, *p* = 0.001. In other words, search was less gamble-wise (suggesting the operation of a strategy foregoing trade-offs, such as the priority heuristic) in difficult than in easy problems. Thus, measures of people's direction of search support the view that properties of the task—here choice difficulty—elicit different choice strategies.

### Summary

The results obtained in Experiment 2 suggest that people recruit different strategies depending on choice difficulty. First, the priority heuristic predicted participants' choices better than cumulative prospect theory (and EV theory) in the context of difficult problems, whereas for easy problems, the pattern was reversed. These results are consistent with findings by Brandstätter et al. (2006, 2008) based on majority choices for data by Mellers et al. (1992) and Erev et al. (2002). Consistent with the findings on the outcome level, on the process level the direction of search proved to be less gamble-wise in difficult than in easy problems. In contrast to overt choices and direction of search, the other process measure, acquisition frequencies, did not reflect the apparent contingency between choice difficulty and strategy use. Moreover, recall that the pattern of acquisition frequencies in Experiment 1 had been inconsistent with both the expectation model and the priority heuristic. How can these findings on acquisition frequencies be interpreted? A pessimistic view would be that this common process measure simply lacks sensitivity to reflect the choice process. Alternatively, frequencies of acquisition could be sensitive to undervalued properties of choice problems and could thus help us to develop a better understanding of the underlying processes. It is to this interpretation that we turn next.

## Acquisition frequencies in risky choice: what do they reflect?

In Experiments 1 and 2, the observed acquisition frequencies proved highest for the maximum outcomes (e.g., Figure 4), a pattern that is not predicted by either the priority heuristic or the expectation model. What underlies this pronounced attention to maximum outcomes?

### Do acquisition frequencies reflect the impact of individual reasons on choice?

Acquisition frequencies are usually interpreted as reflecting the weight (or priority) that a piece of information receives in the decision process (e.g., Payne et al., 1988; Wedell and Senter, 1997). Based on this common interpretation, one should expect strategies assigning the highest priority to the maximum (rather than the minimum) outcomes to be better descriptive models than the priority heuristic or the expectation model. To test this possibility, we examined how well models that use the maximum outcomes as the top-ranked reason are able to predict the participants' individual choices in Experiment 1, and to predict majority choices in the large and diverse set of 260 gamble problems analyzed in Brandstätter et al. (2006). Among the models was a version of the priority heuristic with a modified priority rule (i.e., going through the reasons in the following order: maximum outcome, probability of maximum outcome, and minimum outcome), the maximax heuristic (which considers only the maximum outcomes and takes the gamble with the highest outcome), and two sequential strategies that prioritize maximum outcomes and integrate outcome and probability information^9^. In both test sets, none of these four models predicted choices better than chance. This suggests that the higher acquisition frequencies for the maximum outcomes, relative to the minimum outcomes, are not indicative of their actual weight (or priority) in the choice process. Moreover, prioritizing maximum outcomes would imply risk-seeking for gains and increasing marginal utility within EU theory—consequences for which little empirical evidence exists.

If acquisition frequencies do not seem to reflect the weight given to the individual reasons in the choice process, what do they reflect instead? In the next section, we provide evidence that acquisition frequencies seem to be a function of properties of the choice problem rather than of the choice process.

### Do acquisition frequencies track similarity relations?

Rubinstein (1988) highlighted a property of choice problems that may be critical in the processing of reasons: *similarity* (see also Mellers and Biagini, 1994). He proposed that if the gambles' values on a reason are similar, and those of the remaining reasons are dissimilar *and* all favor the choice of the same gamble, then this gamble will be chosen (see also Leland, 1994). Rubinstein, however, did not define similarity quantitatively. For the purpose of the following analysis, we define similarity as the relative difference between two gambles on a given reason. Specifically, for the similarity of the maximum and minimum outcomes, similarity was calculated as

(4a) $$\Delta_{O^{\max}}=\frac{\left|O_{A}^{\max}-O_{B}^{\max}\right|}{\max\left\{\left|O_{A}^{\max}\right|,\left|O_{B}^{\max}\right|\right\}}$$

and

(4b) $$\Delta_{O^{\min}}=\frac{\left|O_{A}^{\min}-O_{B}^{\min}\right|}{\max\left\{\left|O_{A}^{\min}\right|,\left|O_{B}^{\min}\right|\right\}}.$$

For the probabilities, similarity was calculated as

(5) $$\Delta_{p}=\left|P_{A}-P_{B}\right|.$$

The lower the Δ of a reason, the more similar two gambles are on this reason. We determined for each of the 24 problems in Experiment 1 the average relative acquisition frequency for each reason. In addition, we calculated for each problem the relative differences between the gambles on each reason (i.e., similarity). Are acquisition frequencies related to similarity, thus defined?

Our data indicate some evidence that they are. In the 14 (out of 24) problems in which the maximum outcomes were inspected more frequently than the minimum outcomes, the maximum outcomes were less similar than the minimum outcomes (mean Δs = 0.28 vs. 0.23). Conversely, in the eight problems in which the minimum outcomes were inspected more frequently than the maximum outcomes, the minimum outcomes were less similar than the maximum outcomes (mean Δs = 0.50 vs. 0.06). This suggests that the acquisition frequencies are driven (at least in part) by the similarity structure of the problem: The more dissimilar the corresponding outcome values are, the more frequently they are inspected. Conversely, the more similar they are, the less frequently they are inspected. In fact, the difference between the Δs of the maximum and minimum outcomes were strongly correlated with their difference in acquisition frequencies *r* = 0.49 (*p* = 0.01). These results are consistent with Rubinstein's (1988) hypothesis that similar outcomes are ignored.

To further examine the hypothesis that acquisition frequencies are driven by similarity, we regressed the observed relative acquisition frequencies on similarity, separately for each of the four reasons. (Because the relative differences for the probabilities of the maximum outcomes are identical to those of the minimum outcomes, only one was used in the regression models). The beta weights for the three predictors are reported in Table 3, as well as the *R*^2^s for each of the four regression models. As can be seen, variability in similarity indeed accounted for a considerable amount of variability in the acquisition frequencies across problems. In particular, the similarity on the maximum outcomes was related to the acquisition frequencies of all four reasons. As indicated by the positive regression coefficients in the first column of Table 3, both for the maximum outcomes and the probabilities of the maximum outcomes, there were *more* acquisitions the less similar the maximum outcomes were (i.e., the larger Δ_*O*max_). For the minimum outcomes and the probabilities of the minimum outcomes, by contrast, there were fewer acquisitions the less similar the maximum outcomes were (as indicated by the negative regression coefficients). The similarity of the minimum outcomes showed the same pattern (although with a less pronounced effect). In particular, there were more acquisitions for the minimum outcomes, and fewer acquisitions for the maximum outcomes and the probabilities of the maximum outcomes, the less similar the minimum outcomes were.

**Table 3:** **Results for the similarity analyses of the relative acquisition frequencies in Experiment 1**.

**Dependent variable**	**Predictors**			***R*^2^**
	**Δ^max^_*O*_**	**Δ^min^_*O*_**	**Δ_*p*_**	
*f*_*O*max_	**0.49**	−0.30	−0.14	0.43
*f*_*O*min_	**−0.38**	**0**.**46**	−0.08	0.54
*f*_*P*max_	**0**.**43**	−0.17	0.14	0.34
*f*_*P*min_	**−0.55**	−0.01	0.01	0.30

Shown are standardized regression coefficients when the relative acquisition frequencies (f) for outcomes and probabilities are regressed on how similar the two gambles in a given choice problem are on the four reasons. Note that Δ_P_ is identical for P^max^ and P^min^; therefore, only one number is shown. O^max^, O^min^, P^max^, and P^min^ refer to the maximum and minimum outcomes and their probabilities, respectively. Significant regression coefficients (p = 0.05) are in bold.

Taken together, different process measures seem to reflect different characteristics of the problems. Specifically, direction of search is sensitive to choice difficulty (Experiment 2), whereas acquisition frequencies appear to be a function of similarity. Our results suggest that acquisition frequencies might be a less useful indicator of the weight (or priority) given to the reasons than has been previously assumed [at least in risky choice; see (Wedell and Senter, 1997; Körner et al., 2007)].

## General discussion

We investigated the cognitive processes underlying risky choice using a quantitative model comparison between the priority heuristic and a generic expectation model [focusing on the traditional notion that an expectation is calculated deliberately; for an alternative approach, see Busemeyer and Townsend (1993)]. Previous investigations had concluded from findings showing that people's search processes conflicted with those predicted by the priority heuristic that people instead follow a compensatory process; however, the predictive power of the alternative accounts were not tested against each other based on quantitative process predictions. Here, we conducted such a comparative test; our major findings are as follows: First, people's direction of search was more in line with the predictions derived from the priority heuristic than with those derived from the expectation model (although neither model predicted the observed direction of search perfectly). Second, the cognitive process measures (direction of search, frequency of acquisition) were contingent on properties of the choice task, such as choice difficulty and similarity. When we employed problems in which the priority heuristic and cumulative prospect theory (EV theory) predicted opposite choices (Experiment 2), the priority heuristic captured individual choice and process better in difficult problems, whereas trade-off models did so in easy problems. Therefore, our results support Payne et al.'s (1993) conclusion that “it seems necessary to distinguish multiple decision strategies; one generic strategy with a variation in parameters is not sufficient” (p. 103). An important issue for future inquiry concerns the reasons underlying people's differential strategy use between easy and difficult choice problems. For instance, it could be that a conflict-resolution strategy (i.e., one that avoids trading off conflicting reasons) such as the priority heuristic is employed only if a clearly superior option cannot be identified from an approximate assessment of the gambles' values (for a more extended discussion, see Brandstätter et al., 2008). Third, our analysis of the acquisition frequencies suggests, however, that in order to distinguish between multiple strategies, we need to better understand the extent to which a given process measure in Mouselab and other process-tracing methodologies track properties of the task (e.g., similarity) or of the cognitive process.

### Examining direction of search in risky choice

**Figure 5:**
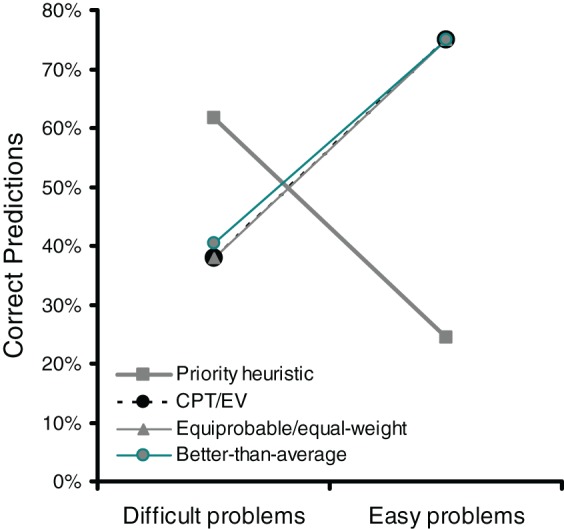
**In easy problems, heuristics that make trade-offs can account for choices equally well as cumulative prospect theory (CPT) and expected value (EV) theory**. Data are from Experiment 2.

Compared with previous process tests of the priority heuristic, we found some striking discrepancies with regard to the absolute degree of gamble-wise and reason-wise search. We know of three published process tests of the priority heuristic that have investigated direction of search using Mouselab or eye tracking (Glöckner and Betsch, 2008a; Johnson et al., 2008; Glöckner and Herbold, 2011). Our results deviate from all three. In these previous experiments, search was considerably more gamble-wise than in ours. For instance, we calculated the SM index from Johnson et al.'s data (two-outcome problems) and found much higher values than ours: 5.1 and 4.5 vs. 0.87 and 0.31 (see Figure 1) for one-reason and three-reason choices, respectively. Why did Glöckner and Betsch (2008a), Johnson et al. (2008), Franco-Watkins and Johnson (2011), and Glöckner and Herbold (2011) find more gamble-wise search than we did? One possibility is that seemingly incidental features of their presentation encouraged more gamble-wise search. Johnson et al. separated the two gambles by a line (see their Figure 1), as did Glöckner and Betsch and Glöckner and Herbold. In addition, the latter two studies as well as Franco-Watkins and Johnson graphically grouped outcome and probability of each branch within a gamble (see Figure 5 in Glöckner and Betsch, or Figure 1 in Franco-Watkins and Johnson). Although we can only speculate at this point, these design features may have nudged participants to search more within a gamble than did our graphical set-up, which avoided such artificial grouping features (Figure 2).

### Why does similarity impact acquisition frequencies?

Our analyses of the role of acquisition frequencies suggest that the more dissimilar the values of gambles on an outcome reason, the more often the outcome (and its probabilities) will be inspected. Why is that? One possible explanation relates acquisition frequencies to memory (rather than informational value). Two very similar values can be “chunked” into one and thus easily kept in memory (e.g., both options have a maximum loss of around 800). With two dissimilar values (e.g., maximum losses of 800 and 1200), however, such chunking does not work and both values need to be stored separately (such memory costs may be amplified somewhat in Mouselab studies, where information acquisition is rather costly). Any forgetting of these values will thus increase the likelihood of re-acquisition of values. This explanation would be consistent with our observation that acquisition frequencies are not predictive of people's choices, but reflect the similarity structure of the choice problem.

### Decision making with and without trade-offs

When trade-offs are made, such as when choice is easy (Experiment 2), how are they made? There are at least two possibilities. First, they could be made via the weighting and summing operations embodied by expectation models. Alternatively, they could be implemented by heuristics that make trade-offs. Consider the first alternative. The simplest version of weighting and summing is EV theory. Alternatively, trade-offs could be made via compensatory but simple processes, such as the equiprobable heuristic, the equal-weight heuristic, or the better-than-average heuristic (see Brandstätter et al., 2006, for a detailed description). Consistent with this possibility, Cokely and Kelley (2009) concluded from their verbal protocol study that “expected-value choices rarely resulted from expected-value calculations” (p. 20). Rather, respondents often reported simple processes such as ordinal comparisons of the values within one reason (e.g., “$900 is a lot more than $125”) or the evaluation of a single probability (e.g., “30% just won't happen”).

In order to evaluate the hypothesis that trade-offs are made based on simple heuristics, in Experiment 2 we tested the ability of various trade-off heuristics to predict individual choice in easy and difficult problems. It emerged that, in easy problems, three of the trade-off heuristics—the equiprobable heuristic, the equal-weight heuristic, and the better-than-average heuristic—reached the highest level of performance (74.9% correct predictions). Figure 5 shows that the three heuristics showed the same performance as cumulative prospect theory and EV theory (the equiprobable heuristic and the equal-weight heuristic always made the same prediction and are therefore depicted together in Figure 5). They predicted choice better than the priority heuristic did when choice was easy, whereas the priority heuristic predicted choice better when choice was difficult (replicating results from Experiment 1). Moreover, note that the equiprobable heuristic, the equal-weight heuristic, and the better-than-average heuristic predicted gamble-wise direction of search—consistent with our finding that direction of search is more gamble-wise in easy than in difficult choice (Experiment 2).

### Limitations

Some possible limitations of our experimental procedure are acknowledged. First, we cannot exclude that labeling in our Mouselab set-up the outcomes as “higher value” and “lower value” might to some extent have influenced people's search direction; future studies could use more neutral labels such as “Outcome 1” and “Outcome 2.” Second, although neither the priority heuristic nor the expectation model predict processes to differ between gains and losses, it should be noted that in Experiment 1 gain and loss problems were not equally distributed across one-, two-, and three-reason choices (see Appendix D). A third possible objection is that in the gamble problems from Mellers et al. (1992) that we used in Experiment 2, one of the outcomes was always zero; this might have led participants to simplify their choice strategy to some extent. Fourth, it has been argued that compared to less obtrusive process-tracing technologies such as eye tracking, Mouselab might encourage more controlled cognitive operations (Glöckner and Betsch, 2008b). However, note that systematic comparisons of Mouselab and eye tracking in risky choice have found little evidence for systematic discrepancies (Lohse and Johnson, 1996; Franco-Watkins and Johnson, 2011). Fifth, in Experiment 1 the estimated choice reliability was relatively low and did not achieve common test-retest reliability standards. Finally, one reviewer pointed out that the use of gamble problems with gambles that have the same expected value might constrain the performance of the expectation models, as in such problems these models would often have to guess. However, prominent expectation models (e.g., prospect theory) were specifically developed to account for systematic choices in problems with gambles having the same expected values [for many examples, see (Kahneman and Tversky, 1979)]. In addition, the expectation models did not have to guess for any of the gamble problems used in our experiments (including those with high choice difficulty).

### Future directions

We have focused on models of risky choice that assume (at least implicitly) a deliberate decision process, as these models have been the key contestants in previous tests of the priority heuristic (e.g., Birnbaum, 2008a; Brandstätter et al., 2008; Glöckner and Betsch, 2008a; Rieger and Wang, 2008). Recently, however, some authors have highlighted the possible contribution of mechanisms involving more automatic information processing in risky choice, such as decision field theory (Johnson and Busemeyer, 2005; Rieskamp, 2008) and parallel constraint satisfaction (Glöckner and Herbold, 2011). In a model comparison investigation based on people's risky choices, for instance, Scheibehenne et al. (2009) found supporting evidence for decision field theory. Despite these encouraging results, it is currently unclear how these models can give rise to several classical empirical regularities such as the fourfold pattern, the common ratio effect, or the common consequence effect—all of which have been critical in the evolution of models of risky choice. Some expectation models (e.g., cumulative prospect theory) and the priority heuristic, by contrast, have been shown to be able to account for these patterns (e.g., Kahneman and Tversky, 1979; Tversky and Fox, 1995; Katsikopoulos and Gigerenzer, 2008). In light of the fact that models of automatic processing seem able to accommodate some aspects of process data that are not predicted by current models assuming more deliberate processes (e.g., Glöckner and Herbold, 2011), future analyses should elaborate how (and whether) these models could give rise to the empirical regularities in choice.

Another important avenue for future research is to develop a better understanding of the considerable heterogeneity in findings on the processes underlying risky choice. In addition to influences of subtle features in the display of information (see Footnote 6), our findings concerning the influence of the similarity structure on process measures indicate that the type of choice problems used might have an as yet neglected impact on the results obtained.

A final task for future investigations is to refine ways to compare heuristics and multiparameter expectation models (e.g., cumulative prospect theory, transfer-of-attention-exchange model) in terms of their ability to predict people's choices. Following previous work, in our analyses we accounted for differences in the number of free parameters between the expectation models and the priority heuristic by using previously published parameter sets for the former; then we compared the models in terms of the percentage of correct predictions. As pointed out in Footnote 4, however, an alternative approach would be to fit the multiparameter models to the data and use more sophisticated model-selection measures, such as BIC or AIC (e.g., Wasserman, 2000), which punish a model depending on the number of free parameters. Because these measures are a function of a model's log-likelihood, applying them to heuristics requires, however, the development of probabilistic versions of the heuristics. Currently it is unclear which of the various choice rules proposed in the literature (i.e., logit, probit, Luce, constant error; see Stott, 2006) is most appropriate for this purpose, also in light of the fact that some heuristics (e.g., the priority heuristic) assume difference thresholds whereas other do not. Rieskamp (2008) has made several suggestions for how to turn deterministic heuristics into probabilistic models and this work might thus serve as a useful starting point.

## Conclusion

How do people make decisions when facing risky prospects? More than 30 years ago, Payne (1973) pointed out that “the earliest research efforts in the area of decision making under risk were conducted by mathematicians and economists. The psychological study of risky decision making has just begun to move away from the influence of these early efforts” (p. 451). Many subsequent studies on the psychology of risky choice using process tracing tools concluded that people rely on heuristic processes rather than on the mathematical principle of expectation (Rosen and Rosenkoetter, 1976; Payne and Braunstein, 1978; Russo and Dosher, 1983; Mann and Ball, 1994; Cokely and Kelley, 2009; Venkatraman et al., 2009; Su et al., 2013). Nevertheless, tests of specific model predictions have been rare. The priority heuristic makes precise process predictions based on the principles of bounded rationality. Recent empirical evidence inconsistent with the predictions of the priority heuristic has prompted several researchers to return to the hypothesis that people rely on compensatory strategies based on the notion of expectation. In this article, we illustrated how a quantitative model comparison approach can be used to evaluate the extent to which people's cognitive processes follow the predictions of the priority heuristic and the expectation model, respectively. Although the process predictions are necessarily based on simplifying assumptions, our results offer, so we believe, some important insights for future comparative tests of quantitative process predictions.

### Conflict of interest statement

The authors declare that the research was conducted in the absence of any commercial or financial relationships that could be construed as a potential conflict of interest.

## Footnotes

^1^In this article we assume a deliberate decision process, as process studies of risky choice as well as most comparative tests of the priority heuristic have focused on models based on deliberate and serial information processing (Payne and Braunstein, 1978; Rieger and Wang, 2008; Birnbaum, 2008a; Glöckner and Betsch, 2008a). However, note that some recent analyses have also considered the possible role of automatic integration processes in risky choice. One such model is decision field theory (DFT; Busemeyer and Townsend, 1993). DFT also generates an expectation-based evaluation, but does so based on a sequential sampling process, rather than by weighting. In a study involving time pressure, Glöckner and Herbold (2011) found some process evidence supporting the predictions of automatic models. As quantitative process predictions—the focus of this article—have not yet been elaborated for these models, however, we refrain from considering them here. We further elaborate on models assuming automatic integration in the General Discussion.

^2^Extending previous process tests of the priority heuristic, our tests include both gain and loss problems (Glöckner and Betsch, 2008a; Johnson et al., 2008; Glöckner and Herbold, 2011, used only gain problems) and problems representing one-, two-, and three-reason choices according to the priority heuristic (Johnson et al. focused on one- and three-reason choices).

^3^Note that in some Mouselab studies on risky choice only the alternatives were labeled, but not the reasons (Johnson et al., 2008), potentially generating a bias toward alternative-wise search. To avoid such a bias, we followed the procedure used in the large majority of Mouselab-type studies on multiattribute choice (e.g., Bettman et al., 1990; Franco-Watkins and Johnson, 2011) and probabilistic inference (e.g., Newell and Shanks, 2003), in which both the alternatives and the reasons were labeled.

^4^An alternative approach would be to estimate the free parameters of the expectation models and to compare all models using a model selection measure such as the Bayesian Information Criterion (BIC), which takes into account the higher flexibility of the expectation models due to the free parameters. We did not use this approach because the calculation of a BIC requires probabilistic model predictions. The heuristics, however, have been proposed as deterministic models and it is currently rather little explored how best to translate them into probabilistic ones (for some suggestions, see Rieskamp, 2008). We further discuss this issue in the General Discussion.

^5^A repeated-measures ANOVA showed that the priority heuristic's performance differed between problem types, *F*_(2, 78)_ = 4.73, *p* = 0.012, and predicted choices better in three-reason (*M* = 70.4, *SD* = 21.8) than in one-reason (*M* = 61.3, *SD* = 13.4) and two-reason choices (*M* = 57.5, *SD* = 20.3). It thus seems unlikely that the larger proportion of one-reason choices among the gamble problems (see Appendix D) gave an advantage to the priority heuristic.

^6^Participants thus made considerably fewer acquisitions than in Johnson et al. (2008), who reported a mean number of 26.7 acquisitions per problem. One possible reason for this discrepancy is that in Johnson et al. participants only had to move the mouse over a box to open it, whereas our participants had to click on the box (but we cannot exclude that other methodological differences between the studies may also have contributed to the difference in the number of acquisitions).

^7^We use bootstrapped confidence intervals of the mean here because the models' RMSDs may not be normally distributed (all conclusions were robust, however, when using parametric methods for statistical inference). For the bootstrapping we used the normal approximation method, based on 1,000 samples, and sampling with replacement.

^8^Additional analyses suggested by an anonymous reviewer showed that, when assuming a very high level of risk aversion, CPT can predict choices in both easy and difficult problems. With the set of parameter values obtained by Erev et al. (2002)—α = β = 0.33 and γ = 0.75 [which according to Fox and Poldrack's (2008), overview are very uncommon], CPT's performance in the difficult problems increased from *M* = 37.9% to 61.7%, matching that of the priority heuristic in this problem set (based on the Erev et al. parameters, CPT's performance in the easy problems was *M* = 68.1%). We see no a priori reason, however, to expect a high level of risk aversion in Experiment 2. Holt and Laury (2002) demonstrated that high levels of risk aversion emerge when large amounts of money are at stake (e.g., $10,000; see also Stott, 2006). The gamble problems in Experiment 2, however, involved only small outcomes.

^9^The sequential models were motivated by the finding reported in a later section (see Table 3) that both the maximum outcomes and their probabilities are examined more if the maximum outcomes are dissimilar; and that minimum outcomes and their probabilities are examined more if the minimum outcomes are dissimilar. The first model starts by examining the maximum outcomes and checks whether their difference exceeds an aspiration level. If it does, the probabilities of the maximum outcomes will also be examined. These two reasons are then combined by tallying. If both reasons favor the same gamble, this gamble will be chosen. If the two reasons favor different gambles, or if the difference between the maximum outcomes does not exceed the aspiration level, the minimum outcomes will be examined following the same logic. That is, if they exceed the aspiration level, the probabilities of the minimum outcomes will be examined. These two reasons are then combined by tallying. If both reasons favor the same gamble, this gamble will be chosen. If the two reasons favor different gambles, one of the gambles will be chosen randomly. The second sequential model differs from the first in that the outcome and the probabilities are combined multiplicatively rather than by tallying.

## Appendix A

**Table A1:** **Derivation of the predicted relative acquisition frequencies**.

**Reason**	**Reading phase**	**Choice phase**				**Choice and reading phases**							
		**Priority heuristic**			**Expectation model**	**Priority heuristic**						**Expectation model**	
		***r* = 1**	***r* = 2**	***r* = 3**		***r* = 1**		***r* = 2**		***r* = 3**			
						**No.**	**%**	**No.**	**%**	**No.**	**%**	**No.**	**%**
Minimum outcome	2	2	2	2	2	4	40	4	33.3	4	28.6	4	25
Maximum outcome	2	0	0	2	2	2	20	2	16.7	4	28.6	4	25
Probability of minimum outcome	2	0	2	2	2	2	20	4	33.3	4	28.6	4	25
Probability of maximum outcome	2	0	0	0	2	2	20	2	16.7	2	14.3	4	25
Total number of acquisitions	8	2	4	6	8	10		12		14		16	

Example: There are eight acquisitions (two for each reason) in the reading phase. In the choice phase, for r = 1, the priority heuristic predicts two further acquisitions, of the minimum outcomes. Thus, across the reading and choice phases, there are a total of 10 acquisitions, in four of which minimum outcomes are examined. r = 1, one-reason choices; r = 2, two-reason choices; r = 3, three-reason choices.

**Table A2:** **Derivation of the predicted transitions (cf. Brandstätter et al., 2008)**.

**Type of transition**	**Reading phase**	**Choice phase**				**Choice and reading phases**							
		**Priority heuristic**			**Expectation model**	**Priority heuristic**						**Expectation model**	
		***r* = 1**	***r* = 2**	***r* = 3**		***r* = 1**		***r* = 2**		***r* = 3**			
						**No.**	**%**	**No.**	**%**	**No.**	**%**	**No.**	**%**
Outcome-probability	4	0	1	1	4	4	50	5	50	5	42	8	57
Other within-gamble	2	0	0	1	2	4	25	2	20	3	25	4	29
Within-reason	1	1	2	3	1	2	25	3	30	4	33	2	14
Other	0	0	0	0	0	0	0	0	0	0	0	0	0
Total number of transitions	7	1	3	5	7	8		10		12		14	

r = 1, one-reason choices; r = 2, two-reason choices; r = 3, three-reason choices.

## Appendix B

### Derivation of predicted transitions based on alternative assumptions about the reading phase

Because predictions about transition probabilities are sensitive to assumptions about the reading phase, we also calculated the predictions using an alternative reading order. Specifically, we assumed that rather than reading within gambles [as assumed by Brandstätter et al. (2008)], people follow the natural reading direction (i.e., from left to right). Table B1 shows the predicted number of transitions for the outcome-probability, other within-gamble transitions, and within-reason transitions. Table B2 shows the resulting predicted SM values, which diverge considerably more from the data than do the predictions reported in Table 2, for both the priority heuristic and the expectation model. This suggests that people are more likely to read within gambles than according to the natural reading direction.

**Table B1:** **Derivation of the predicted transitions based on alternative assumptions for the reading phase**.

**Types of transitions**	**Reading phase**	**Choice phase**				**Choice and reading phases**							
		**Priority heuristic**			**Expectation model**	**Priority heuristic**						**Expectation model**	
		***r* = 1**	***r* = 2**	***r* = 3**		***r* = 1**		***r* = 2**		***r* = 3**			
						**No.**	**%**	**No.**	**%**	**No.**	**%**	**No.**	**%**
Outcome-probability	2 (0/4)	0	1	1	4	2	25	3	30	3	25	6	42.9
Other within-gamble	1 (0/2)	0	0	1	2	1	12.5	1	10	2	16.7	3	21.4
Within-reason	2 (4/0)	1	2	3	1	3	37.5	4	40	5	41.7	3	21.4
Other	2 (3/1)	0	0	0	0	2	25	2	20	2	16.7	2	14.3
Total number of transitions	7	1	3	5	7	8		10		12		14	

r = 1, one-reason choices; r = 2, two-reason choices; r = 3, three-reason choices. The second column shows the expected number of transitions averaged across both set-up orientations, the numbers in the brackets show the expected number of transitions separately for the vertical and horizontal set-ups, respectively.

**Table B2:** **Predicted SM index based on alternative assumptions for the reading phase, separately for one-reason (*r* = 1), two-reason (*r* = 2), and three-reason (*r* = 3) choices**.

	***r* = 1**	***r* = 2**	***r* = 3**
**SM INDEX**			
**Predictions**			
Priority heuristic	−0.38	−0.38	−0.38
Expectation model	6.11	6.11	6.11

## Appendix C

### Further details on the SM index

The SM index is a function of the difference between the number of gamble-wise and the number of reason-wise transitions. *Reason-wise* transitions were defined as transitions between boxes belonging to the same reason; *gamble-wise* transitions were defined as transitions between boxes belonging to outcomes and those belonging to the outcomes' probabilities, as well as other transitions between reasons within a gamble. Note from Equation 1 that transitions that occur both between gambles and between different reasons are not specifically quantified, although they are included in the total number of transitions, *N*. The percentage of transitions of this type was, on average, 20.7% in Experiment 1 and 19.1% in Experiment 2. As pointed out by Payne and Bettman (1994), the SM index is sensitive not only to the sequence of information search, but also to the number of pieces of information acquired. To address this problem and also to ensure comparability with the model predictions (which were based on percentages; see Table 2), the empirical SM values were normalized such that *N* was set to 100. The values used for *n*_gamble_ and *n*_reason_ (for each participant) were thus the percentages of gamble-wise and reason-wise transitions, respectively, of all transitions (= 100%).

## Appendix D

**Table D1:** **Gamble problems used in Experiment 1 and the obtained choice proportions**.

**Problem type**	**Gamble A**	**Gamble B**	**EV ratio**	**Choice proportions for gamble A (in %)**	**Source**
*r* = 1	2000, 0.6; 500, 0.4	2000, 0.4; 1000, 0.6	1^a^	42.5	BGH
	4000, 0.2; 2000, 0.8	3000, 0.7; 1000, 0.3	1^a^	35	BGH
	800, 0.8; 500, 0.2	820, 0.6; 600, 0.4	1.01	50	PHGB
	5000, 0.7; 100, 0.3	5000, 0.65; 1000, 0.35	1.02	37.5	PHGB
	−500, 0.4; −2000, 0.6	−1000, 0.6; −2000, 0.4	1^a^	60	BGH
	−2000, 0.7; −5000, 0.3	−2800, 0.9; −4800, 0.1	1.04	62.5	PHGB
	−500, 0.2; −800, 0.8	−600, 0.4; −820, 0.6	1.01	47.5	PHGB
	−50, 0.3; −3500, 0.7	−600, 0.35; −3400, 0.65	1.02	72.5	PHGB
	−100, 0.3; −5000, 0.7	−1000, 0.35; −5000, 0.65	1.02	82.5	PHGB
	−50, 0.1; −900, 0.9	−400, 0.15; −880, 0.85	1.01	70	PHGB
	−2000, 0.6; −2350, 0.4	−1700, 0.55; −2500, 0.45	1.04	37.5	PHGB
	−150, 0.7; −2500, 0.3	−650, 0.9; −2400, 0.1	1.04	72.5	PHGB
*r* = 2	2000, 0.5; 0, 0.5	4000, 0.2; 300, 0.8	1.04	42.5	PHGB
	1600, 0.3; 1000, 0.7	1300, 0.5; 1000, 0.5	1.03	42.5	PHGB
	1800, 0.2; 200, 0.8	1000, 0.4; 200, 0.6	1	42.5	PHGB
	6000, 0.45; 0, 0.55	3000, 0.9; 0, 0.1	1^a^	20	KT
	−1000, 0.7; −1600, 0.3	−1000, 0.5; −1300, 0.5	1.03	37.5	PHGB
	0, 0.55; −6000, 0.45	0, 0.1; −3000, 0.9	1^a^	70	KT
*r* = 3	6000, 0.3; 2500, 0.7	8200, 0.25; 2000, 0.75	1^a^	20	BGH
	3000, 0.4; 2000, 0.6	3600, 0.35; 1750, 0.65	1.001^a^	52.5	BGH
	6000, 0.001; 0, 0.999	3000, 0.002; 0, 0.998	1^a^	72.5	KT
	4000, 0.2; 0, 0.8	3000, 0.25; 0, 0.75	1.07^a^	70	KT
	0, 0.8; −4000, 0.2	0, 0.75; −3000, 0.25	1.07^a^	27.5	KT
	0, 0.999; −6000, 0.001	0, 0.998; −3000, 0.002	1^a^	20	KT

r = 1, one-reason choices; r = 2, two-reason choices; r = 3, three-reason choices. The last column indicates whether the gamble problem is from Brandstätter et al. (2006) (BGH), or from Kahneman and Tversky (1979) (KT), or were created for the current article (PHGB).

a Included in the paper-and-pencil task in Experiment 1.

**Table D2:** **Gamble problems used in Experiment 2 and the obtained choice proportions**.

**Choice difficulty, domain**	**Gamble A**	**Gamble B**	**EV ratio**	**Choice proportions for gamble A (in %)**
Easy, gains	3, 0.17; 0, 0.83	56.7, 0.05; 0, 0.95	5.6	32.5
	3, 0.29; 0, 0.71	56.7, 0.09; 0, 0.91	5.9	51.3
	56.7, 0.05; 0, 0.95	3, 0.17; 0, 0.83	5.6	70.0
	56.7, 0.09; 0, 0.91	3, 0.29; 0, 0.71	5.9	55.0
	5.4, 0.52; 0, 0.48	56.7, 0.29; 0, 0.71	5.9	15.0
	3, 0.94; 0, 0.06	56.7, 0.29; 0, 0.71	5.8	32.5
	31.5, 0.29; 0, 0.71	3, 0.52; 0, 0.48	5.9	89.7
	56.7, 0.29; 0, 0.71	5.4, 0.52; 0, 0.48	5.9	82.5
	3, 0.94; 0, 0.06	31.5, 0.52; 0, 0.48	5.8	10.0
	5.4, 0.94; 0, 0.06	56.7, 0.52; 0, 0.48	5.8	22.5
	31.5, 0.52; 0, 0.48	3, 0.94; 0, 0.06	5.8	72.5
	56.7, 0.52; 0, 0.48	5.4, 0.94; 0, 0.06	5.8	77.5
Easy, losses	0, 0.83; −3, 0.17	0, 0.95; −56.7, 0.05	5.6	61.6
	0, 0.71; −3, 0.29	0, 0.91; −56.7, 0.09	5.9	57.5
	0, 0.95; −56.7, 0.05	0, 0.83; −3, 0.17	5.6	32.5
	0, 0.91; −56.7, 0.09	0, 0.71; −3, 0.29	5.9	27.5
	0, 0.48; −3, 0.52	0, 0.71; −31.5, 0.29	5.9	82.1
	0, 0.06; −3, 0.94	0, 0.71; −56.7, 0.29	5.8	80.0
	0, 0.71; −31.5, 0.29	0, 0.48; −3, 0.52	5.9	18.0
	0, 0.71; −56.7, 0.29	0, 0.48; −5.4, 0.52	5.9	17.5
	0, 0.06; −3, 0.94	0, 0.48; −31.5, 0.52	5.8	87.5
	0, 0.06; −5.4, 0.94	0, 0.48; −56.7, 0.52	5.8	80.0
	0, 0.71; −56.7, 0.29	0, 0.06; −3, 0.94	5.8	15.4
	0, 0.48; −31.5, 0.52	0, 0.06; −3, 0.94	5.8	12.5
Difficult, gains	17.5, 0.52; 0, 0.48	56.7, 0.17; 0, 0.83	1.1	72.5
	9.7, 0.52; 0, 0.48	31.5, 0.17; 0, 0.83	1.1	77.5
	5.4, 0.29; 0, 0.71	9.7, 0.17; 0, 0.83	1.1	57.5
	31.5, 0.29; 0, 0.71	56.7, 0.17; 0, 0.83	1.1	70.0
	3, 0.29; 0, 0.71	5.4, 0.17; 0, 0.83	1.1	67.5
	3, 0.52; 0, 0.48	9.7, 0.17; 0, 0.83	1.1	65.0
	17.5, 0.17; 0, 0.83	3, 0.94; 0, 0.06	1.1	22.5
	9.7, 0.17; 0, 0.83	5.4, 0.29; 0, 0.71	1.1	35.0
	56.7, 0.17; 0, 0.83	17.5, 0.52; 0, 0.48	1.1	27.5
	9.7, 0.17; 0, 0.83	3, 0.52; 0, 0.48	1.1	23.1
	5.4, 0.17; 0, 0.83	3, 0.29; 0, 0.71	1.1	30.0
	31.5, 0.17; 0, 0.83	5.4, 0.94; 0, 0.06	1.1	20.0
Difficult, losses	0, 0.48; −3, 0.52	0, 0.83; −9.7, 0.17	1.1	43.6
	0, 0.71; −5.4, 0.29	0, 0.83; −9.7, 0.17	1.1	55.0
	0, 0.48; −17.5, 0.52	0, 0.83; −56.7, 0.17	1.1	45.0
	0, 0.71; −9.7, 0.29	0, 0.83; −17.5, 0.17	1.1	61.5
	0, 0.06; −5.4, 0.94	0, 0.83; −31.5, 0.17	1.1	37.5
	0, 0.06; −3, 0.94	0, 0.83; −17.5, 0.17	1.1	40.0
	0, 0.83; −9.7, 0.17	0, 0.71; −5.4, 0.29	1.1	42.5
	0, 0.83; −17.5, 0.17	0, 0.48; −5.4, 0.52	1.1	65.0
	0, 0.83; −17.5, 0.17	0, 0.71; −9.7, 0.29	1.1	42.5
	0, 0.83; −56.7, 0.17	0, 0.48; −17.5, 0.52	1.1	61.5
	0, 0.83; −5.4, 0.17	0, 0.71; −3, 0.29	1.1	57.5
	0, 0.83; −31.5, 0.17	0, 0.48; −9.7, 0.52	1.1	47.5

As an illustration of the priority heuristic and cumulative prospect theory predicting opposite choices in these gamble problems, take the first problem, A (3, 0.17; 0, 0.83) vs. B (56.7, 0.05; 0, 0.95). The priority heuristic would base a choice on the probability of the minimum outcomes (as the minimum outcomes do not discriminate) and predict the choice of gamble A because it has the lower probability of yielding the minimum outcome. Cumulative prospect theory (based, for instance, on the parameter set by Tversky and Kahneman, 1992) would assign a subjective valuation of 0.634 to gamble A and a subjective valuation of 4.597 to gamble B. Therefore, cumulative prospect theory predicts the choice of gamble B.

## Appendix E

### Instructions used in Experiment 1

Dear participant,

Thank you very much for taking part in this study. We are interested in how people make decisions between options with risky outcomes. We have constructed a task in which you are asked to choose between two gambles. Each gamble has two possible outcomes. Each outcome occurs with a certain probability.

Each gamble is thus characterized by two types of information:

- the possible outcomes,
- the probability that each outcome will occur.

You will be presented with several trials, each involving two gambles. Your task is to decide which of the two gambles you would prefer. Here is an example:

**Table:**

Gamble A:	You win	€10 with a probability (*p*) of 0.2 or
		€1 with a probability (*p*) of 0.8
Gamble B:	You win	€4 with a probability (*p*) of 0.4 or
		€3 with a probability (*p*) of 0.6

Each gamble thus involves the chance to obtain two different, mutually exclusive outcomes, and each outcome is characterized by different amounts to win or lose (note that within each gamble you can only win *or* lose). The outcomes occur with certain probabilities (varying between 0.01 und 0.99). Each gamble is thus characterized by two possible outcomes—a larger amount or a smaller amount—as well as probability (indicated by “p”) that the outcome will occur. Within each gamble, the probabilities of the larger and the smaller amount always add up to 1.

The information about the gambles (i.e., their outcomes and their probabilities) is hidden; you have to click on the information box to uncover it. The information remains visible as long as you press the mouse button.

In other words, you have to search for the information you need to make a decision. Once you have acquired sufficient information to make a decision, please press either “Choose A” if you prefer gamble A or “Choose B” if you prefer gamble B. You will be presented with a total of 33 trials with different gambles. At the end of the experiment, one of the gamble problems will be selected randomly and the gamble you chose will be played out. You will win (or lose) an amount proportional to the payoff obtained.

Please turn to the experimenter for further assistance.

## Appendix F

### Analysis of participants' choices in Experiment 1

To derive the choice predictions for cumulative prospect theory (CPT), we used the two sets of parameter estimates from Tversky and Kahneman (1992) and Lopes and Oden (1999), which represent the values obtained in other studies rather well (e.g., Glöckner and Pachur, 2012; for an overview, see Fox and Poldrack, 2008). For security-potential/aspiration theory, we used the parameter estimates from Lopes and Oden (1999); for the transfer-of-attention-exchange model, we used the parameters proposed by Birnbaum (2004). We acknowledge that constraining the flexibility of multiparameter models by using parameters obtained in other studies—though employed not only in risky choice (e.g., Brandstätter et al., 2006; Birnbaum, 2008a; Glöckner and Betsch, 2008a) but also in other areas of cognitive science, such as memory (e.g., Anderson et al., 2004; Oberauer and Lewandowsky, 2008)—may underestimate the descriptive accuracy of the expectation models as compared to when they are fit to data. The mean (across participants) percentage of correct predictions of the different models is reported in Table F1.

**Table F1:** **How well do expectation models and heuristics capture participants' choices in Experiment 1?**.

	**Proportion of correct predictions (*M*)**
**EXPECTATION MODELS**	
Cumulative prospect theory (LO)	0.51
Cumulative prospect theory (TK)	0.59
Security-potential/aspiration theory	0.54
Transfer-of-attention-exchange model	0.57
**HEURISTICS**	
Priority heuristic	0.63
Equiprobable	0.59
Equal-weight	0.59
Minimax	0.47
Maximax	0.56
Better-than-average	0.50
Most-likely	0.51
Lexicographic	0.57
Least-likely	0.41
Probable	0.50
Tallying	0.41

The table shows the average (across participants) proportion of correct predictions for each model. LO, using parameter estimates reported by Lopes and Oden (1999); TK, using parameter estimates reported by Tversky and Kahneman (1992).
